# Synthesis of Core–Double Shell Nylon-ZnO/Polypyrrole Electrospun Nanofibers

**DOI:** 10.3390/nano10112241

**Published:** 2020-11-12

**Authors:** Mihaela Beregoi, Nicoleta Preda, Andreea Costas, Monica Enculescu, Raluca Florentina Negrea, Horia Iovu, Ionut Enculescu

**Affiliations:** 1 Laboratory of Multifunctional Materials and Structures, National Institute of Materials Physics, Atomistilor 405A, 077125 Magurele, Romania; nicol@infim.ro (N.P.); andreea.costas@infim.ro (A.C.); mdatcu@infim.ro (M.E.); raluca.negrea@infim.ro (R.F.N.); 2 Advanced Polymer Materials Group, Faculty of Applied Chemistry and Materials Science, University Politehnica of Bucharest, Gheorghe Polizu 1-7, 060042 Bucharest, Romania; iovu@tsocm.pub.ro

**Keywords:** polypyrrole, zinc oxide, nanofiber, electrospinning, electrodeposition, sol–gel, core–double shell

## Abstract

Core–double shell nylon-ZnO/polypyrrole electrospun nanofibers were fabricated by combining three straightforward methods (electrospinning, sol–gel synthesis and electrodeposition). The hybrid fibrous organic–inorganic nanocomposite was obtained starting from freestanding nylon 6/6 nanofibers obtained through electrospinning. Nylon meshes were functionalized with a very thin, continuous ZnO film by a sol–gel process and thermally treated in order to increase its crystallinity. Further, the ZnO coated networks were used as a working electrode for the electrochemical deposition of a very thin, homogenous polypyrrole layer. X-ray diffraction measurements were employed for characterizing the ZnO structures while spectroscopic techniques such as FTIR and Raman were employed for describing the polypyrrole layer. An elemental analysis was performed through X-ray microanalysis, confirming the expected double shell structure. A detailed micromorphological characterization through FESEM and TEM assays evidenced the deposition of both organic and inorganic layers. Highly transparent, flexible due to the presence of the polymer core and embedding a semiconducting heterojunction, such materials can be easily tailored and integrated in functional platforms with a wide range of applications.

## 1. Introduction

Developing novel flexible materials with versatile properties using industrial scalable, low cost and straightforward fabrication methods is a major priority. Hybrid organic–inorganic composites are a class of materials that received a great interest due to the embedment of both compound features. Many hybrid composite configurations were reported in the literature [1,2,3,4,5,6,7,8], the fibrous nanomorphology being an attractive possibility [9,10].

Electrospinning is a fashionable technique intensively utilized to fabricate fiber-based composites with controlled features, compositions and functions [11,12,13]. When pumping a polymer solution through a syringe needle while applying a high intensity electric field between this needle and the collector, freestanding, flexible micro- or nanofiber networks can be obtained. Such meshes can be used either as prepared [14,15,16] or functionalized with various organic, inorganic nanostructures or both. One approach is covering them with thin films in order to obtain core–shell or core–double shell materials with applications as actuators [17,18,19], sensors [17,18,20,21,22], supercapacitors [9,23,24], tissue scaffolds [9,25,26], etc.

Zinc oxide (ZnO) is an n-type semiconductor involved in many material designs, especially when morphologically tailored nanostructures are required. The possibility of utilizing multiple preparation routes in order to obtain various shaped nanostructures characteristics that range from biocompatibility to interesting optoelectronic properties, make this material quite interesting for multiple applications. A lot of chemical or electrochemical preparation ways can be identified for obtaining ZnO thin films or nanostructures. Sol–gel synthesis is one such simple ZnO preparation method that uses inexpensive precursors, being an appropriate technique for covering large areas of materials like electrospun nets [27,28].

Polypyrrole (PPy) is the most used π conjugated conducting polymer with a p-type semiconductor behavior. Properties such as the structure and morphology, conductivity and stability strongly depend on preparation methods and dopants. The material can be rapidly synthesized starting from the monomer by chemical oxidation or electropolymerization. The second method is preferred due to the possibility of a good control of the deposit’s characteristics and improved conductivity. PPy in a web-like configuration is quite difficult to obtain due to its highly insolubility in common solvents and having low mechanical stability. So, in order to obtain fibrous PPy structures by methods like electrospinning it is necessary to combine it with carrier polymers [29,30,31], leading to inferior electrical characteristics.

The combination of ZnO with PPy was intensively exploited for obtaining materials with improved properties, especially for generating a p–n organic–inorganic heterojunction. Several papers describe the fabrication of electrospun fiber meshes based on ZnO and PPy, the approaches consisting of decorating the PPy based fibers with ZnO nanoparticles [32] or vice versa [7,33,34] and in situ polymerization of PPy in the presence of ZnO [35]. On our knowledge, such core–double shell fibrous material fabricated mixing the ZnO chemical and PPy electrochemical synthesis have not been reported in the scientific literature so far.

The present work describes the fabrication and characterization of a hybrid organic–inorganic nanocomposite based on electrospun fiber meshes used as flexible substrates functionalized with very thin ZnO and PPy films. The proposed fabrication procedure involves the electrospinning of nylon 6/6 in order to obtain freestanding nanofibers, coating them with a ZnO layer by a sol–gel process and electrochemically depositing a continuous PPy film on the ZnO shell. The core–double shell nylon-ZnO/polypyrrole nanofibers were thoroughly micromorphological, structural and optical characterized, and the formation of ZnO with a hexagonal wurtzite structure and of PPy being demonstrated. Fabrication conditions were chosen as to preserve the high specific surface of the initial electrospun meshes. The described method has multiple advantages: being versatile allows fast fabrication using low cost precursors; the material’s features can be further tailored by changing some experimental parameters in accordance with the targeted application; enables obtaining of a flexible material (given by the presence of nylon core) with high transparency (by optimizing fiber density), which possess the characteristic properties of ZnO and PPy. Due to the material’s properties described ahead and preparation method versatility, such kind of structures can be easily integrated in wearable sensors, actuators, intelligent clothes or other outstanding devices.

## 2. Materials and Methods

### 2.1. Materials

All preparation materials, namely nylon 6/6 (pellets, Sigma Aldrich, St. Louis, MO, USA; CAS number 32131-17-2), formic acid (ACS reagent, ≥88.0%, Sigma Aldrich, St. Louis, MO, USA; CAS number 64-18-6), zinc acetate dihydrate (Zn(CH_3_COOH)_2_·2H_2_O, ACS reagent, ≥98%, Sigma Aldrich, St. Louis, MO, USA; CAS number 5970-45-6), ethanol absolute (puriss. p.a., absolute, ≥99.8%, Sigma Aldrich, St. Louis, MO, USA; CAS number 64-17-5), lithium perchlorate (LiClO_4_, battery grade, dry, 99.99% metals basis, Aldrich, St. Louis, MO, USA; CAS number 7791-03-9), pyrrole (for synthesis, Merck, Darmstadt, Germany; CAS number 109-97-7), and acetonitrile (for HPLC, gradient grade, ≥99.9%, Honeywell, Charlotte, NC, USA; CAS number 75-05-8) were used as received.

### 2.2. Fabrication of Core–Double Shell Nylon-ZnO/PPy Electrospun Nanofibers

Nylon 6/6 nanofibers (material labeled as nylon) were prepared by electrospinning a precursor solution of 10% (*w/v*) nylon 6/6 in formic acid. The experimental process is a “classic” one, namely: the solution was loaded in a syringe with a 0.4 mm inner diameter metallic needle and pumped (using a New Era Pump System NE-1000, New Era Pump System, New York, NY, USA) with a feed rate of 0.5 mL/h; the electrospinning was performed by applying 25 kV (utilizing a Spelmann SE300 high voltage source, Spellman High Voltage Electronics Corporation, New York, NY, USA) between the needle and the collector (a copper wire frame); the collecting time of the nanofibers was chosen such that the meshes have an optimum fiber density; the distance between the syringe tip and collector was ~15 cm and the nanofibers were collected on copper wire frames as freestanding nets; the electrospinning setup was placed into a plexiglass box in order to keep a constant relative humidity at ~20% and a process temperature of ~23 °C.

Further, the nanofibers were functionalized with a very thin ZnO layer by a sol–gel process, making 15 consecutive cycles of immersing/extracting meshes into/from the precursor solution (0.1 M Zn(CH_3_COOH)_2_·2H_2_O in ethanol), letting them dry between the cycles. In the end, the ZnO coated meshes were rinsed with ethanol for remove any precursor traces. The prepared core–shell nanocomposite was thermally treated in the oven for 3 h at 200 °C in order to improve the crystallinity of ZnO (material labeled as Nylon-ZnO), this temperature being selected so that the nylon core is preserved.

**Scheme 1:**
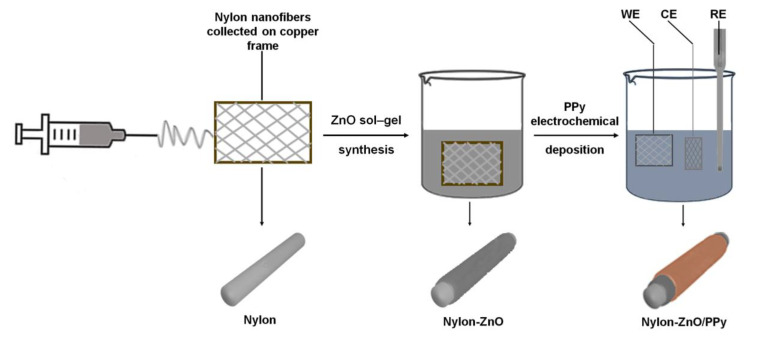
Schematic representation of the nylon-ZnO/PPy fabrication procedure.

The freestanding ZnO coated webs attached on copper wire frames were transferred onto stainless steel frames by mechanical gripping in order to have a good electrical contact for PPy electrochemical deposition. The PPy synthesis was performed using Nylon-ZnO nets attached on these frames as a working electrode (WE), a platinum mesh as a counter electrode (CE) and a commercial saturated calomel electrode (SCE) as reference. The deposition solution was consisted in 0.2 M pyrrole and 0.1 M LiClO_4_ in acetonitrile. A potential of +0.70 V (vs. SCE) was applied for 5 min, the deposition time being selected so that the PPy covered only the nanofibers as a thin, homogenous film (not embedding them into a thick PPy layer, diminishing the high active surface of the electrospun networks). After the deposition process, the core–double shell nanocomposite was rinsed with acetonitrile for removing the unreacted monomer and dopant (material labeled as Nylon-ZnO/PPy). The PPy electrodeposition was performed using a Parstat 2273 Princeton Applied Research potentiostat. All fabrication steps were schematically represented in Scheme 1 and it is worth mentioning that they can be tailored in order to obtain the best material configuration for targeted application.

### 2.3. Characterization

All prepared materials were morphologically characterized using a Zeiss Merlin compact field emission scanning electron microscope (FESEM) (Carl Zeiss, Oberkochen, Germany). The transmission electron (TEM) and high resolution transmission electron (HRTEM) investigations were performed on a Cs probe-corrected JEM ARM 200F microscope (Jeol, Tokyo, Japan) operated at 200 kV in order to estimate the layer thickness. The Zeiss EVO 50XVP scanning electron microscope (Carl Zeiss, Oberkochen, Germany) equipped with an energy dispersive X-ray analysis (EDX) QUANTAX Bruker 200 accessory (Bruker, Billerica, MA, USA) was employed for establishing the elemental composition of fabricated materials. The optical properties were investigated registering the reflectance spectra utilizing a PerkinElmer Lambda 45 UV–VIS spectrophotometer (PerkinElmer, Inc., Waltham, MA, USA) equipped with an integrating sphere. The formation of ZnO was emphasized by X-ray diffraction (XRD) using a Bruker AXS D8 Advance instrument (Bruker, Billerica, MA, USA) with Cu Kα radiation (λ = 0.154 nm) operating at 40 kV and 40 mA. The synthesis of PPy was demonstrated by Fourier transfer infrared spectroscopy (FTIR) using a PerkinElmer Spotlight Spectrum 100 spectrometer (PerkinElmer, Inc., Waltham, MA, USA) and Raman spectroscopy employing a BRUKER-RFS27 FT-Raman spectrometer (Bruker Optik GmbH, Bremen, Germany) with 633 nm/17 mW and 325 nm/25 mW laser kits. For a rigorous characterization, the analysis was also performed for as spun nylon mesh. For all subsequent measurements, nylon, nylon-ZnO and nylon-ZnO/PPy meshes were peeled off from copper or stainless steel frames and placed on Si/SiO_2_ substrates.

## 3. Results and Discussion

**Figure 1:**
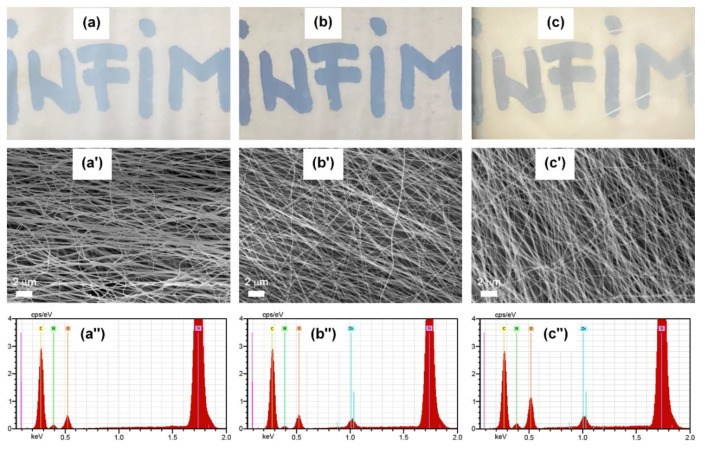
Photographs, FESEM images and energy dispersive X-ray analysis (EDX) spectra of (**a**,**a’**,**a’’**) nylon, (**b**,**b’**,**b’’**) nylon-ZnO and (**c**,**c’**,**c’’**) nylon-ZnO/PPy, respectively.

The chosen fiber density was in a range that leads to transparent webs, as can be observed from the photographs of nylon, nylon-ZnO and nylon-ZnO/PPy presented in Figure 1a–c. No modification of color appearance of nylon-ZnO when it is compared with nylon can be observed, because both nylon and ZnO were unstained, while the mesh became light brown after PPy coverage due to its brown/black color, which could be a confirmation of PPy deposition. Further, the FESEM images at low magnification of nylon, nylon-ZnO and Nylon-ZnO/PPy depicted in Figure 1a’–c’) evidence the partially alignment of nanofibers. A uniform fibrous aspect was present for all the samples, with no ZnO or PPy growth between polymer nanofibers. This means that both compounds were deposited only on the nanofibers, preserving in this way the morphology and consequently the high active area of the electrospun networks. As was expected, the EDX spectra of all prepared materials (Figure 1c’–c’’) show the presence of C, N and O from nylon 6/6 and PPy structures, O appearing also from ZnO, while Zn was detected only in the case of nylon-ZnO and nylon-ZnO/PPy. These prove the existence of ZnO in both materials, meaning that ZnO remains on the nanofibers after PPy electrodeposition.

**Figure 2:**
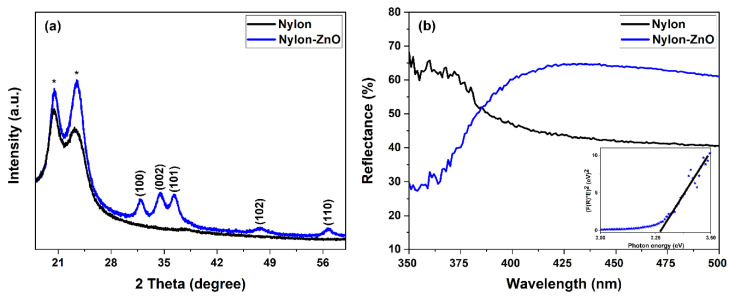
(**a**) XRD patterns and (**b**) reflectance spectra of the fabricated networks.

The ZnO structure was analyzed by registering the XRD diffractograms of nylon and nylon-ZnO, which are plotted in Figure 2a. The XRD pattern of nylon net exhibited two broad peaks at 20.5° and 23.5° (see * from Figure 2a) related to the diffraction of (100) and (010, 110) crystal planes. This result indicates that the nylon was in the α state, the registered peaks being attributed to the distance between the hydrogen-bonded chains and the separation of hydrogen-bonded sheets, respectively [36,37]. Likewise, the nylon-ZnO diffractogram revealed a nylon signature and diffraction peaks at 2θ: 31.9°, 34.5°, 36.3°, 47.6° and 56.7° assigned to (100), (002), (101), (102) and (110) planes of the hexagonal wurtzite phase of ZnO (ICDD 00-035-1451).

The optical properties of nylon and nylon-ZnO were first analyzed recording the reflectance spectra, displayed in Figure 2b. After the ZnO functionalization and annealing, a decrease in reflectance at aproximately 400 nm can be observed for nylon-ZnO, linked to band to band transition in ZnO structure [38]. For this, a band gap of ~3.3 eV was estimated by plotting the Kubelka–Munk function ([F(R)·E]^2^) versus photon energy (E; Figure 2b inset), with F(R) = (1 − R)^2^/2R where R is the experimental diffuse reflectance. The band gap value is in agreement with other previous results [39].

**Figure 3:**
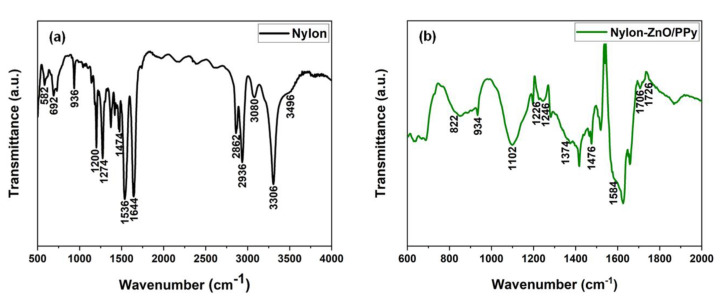
FTIR spectra of (**a**) as spun nanofibers and (**b**) PPy/ZnO coated meshes.

The as spun nylon and nylon-ZnO/PPy meshes were further characterized by FTIR and Raman spectroscopy in order to demonstrate the formation of PPy on nylon-ZnO. Thus, in the nylon FTIR spectrum shown in Figure 3a can be noticed the characteristic absorption peaks of nylon 6/6 [36,40], as follows: the bands at 3496, 3306 and 1474 cm^−1^ correspond to the N–H stretching in amide I and II and N–H deformation, respectively; the peak at 3080 cm^−1^ was assigned to the asymmetric C–H stretching vibrations, while those at 2936, 2862 and 1200 cm^−1^ appeared due to the asymmetric and symmetric stretching and twisting vibrations of CH_2_; the bands at 1644, 1536 and 1274 cm^−1^ were correlated with the stretching vibations of amide I, II and III; the absorption peaks at 936, 692 and 582 cm^−1^ were attributed to the stretching, bending and deformation of C–C bonds.

In the FTIR spectrum of nylon-ZnO/PPy (Figure 3b) only the absorption peaks typical of PPy can be observed because the measurements were performed using a nylon sample as the background. Accordingly, the formation of PPy is confirmed by the presence of absorption bands placed: at 1726 cm^−1^, which corresponds to vibration modes of the C=O bond due to the formation of a slightly overoxidized polypyrrole; at 1706 and 1584 cm^−1^ related to the stretching vibrations of C=C and C–C of pyrrole rings; at 1476, 1374 and 1226 cm^−1^ assigned to the stretching vibrations of C–N bonds; as well, at 1246 cm^−1^ associated with the in plane vibrational modes of C–H, while those at 816 and 954 cm^−1^ are specific to out-of-plane stretching vibrations of C–H; at 1102 cm^−1^ being correlated with the N–H in plane deformation. All these results are in good agreement with those reported in the scientific literature [41].

**Figure 4:**
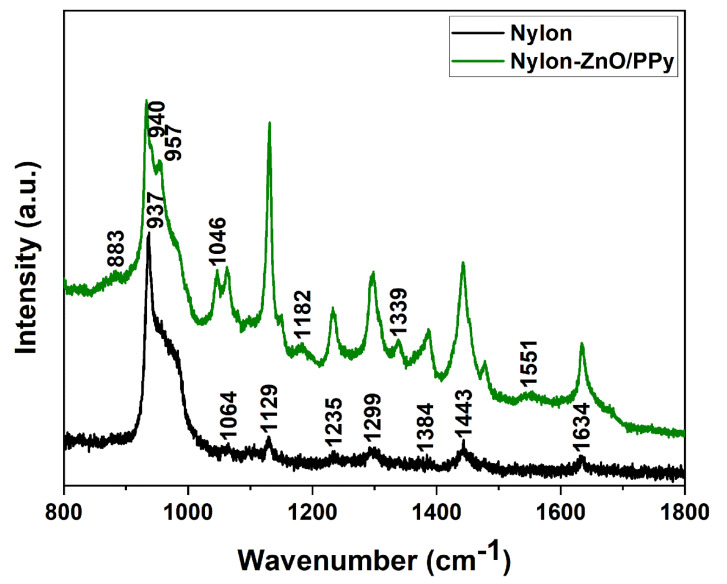
Raman spectra of as spun nanofibers and PPy/ZnO coated meshes.

Complementary results were obtained by registering the Raman spectra of nylon and nylon-ZnO/PPy presented in Figure 4. The typical infrared signature of nylon 6/6 was evidenced through the presence of: the absorption peaks at 1634 and 1443 cm^−1^, which correspond to the vibrational modes of amide I and II; the bands at 1384 and 1299 cm^−1^ that are assigned to the wagging and twisting vibration of CH_2_; the peaks at 1235 cm^−1^ related to N–H wagging vibration modes; the peak at 1129 and 1064 cm^−1^ attributed to the stretching vibration of the C–C bond from trans-conformers of aliphatic chains, the absence of peak at 1080 cm^−1^ denoting that all nylon chains are in trans-configuration; the band at 937 cm^−1^ specific to the C–C–O stretching modes [42,43]. In comparison, the Raman spectrum of nylon-ZnO/PPy includes the fingerprint of nylon 6/6 and the PPy vibrational modes as follows: the band at 1551 cm^−1^ was assigned to C=C stretching vibration and that at 1339 cm^−1^ arose from PPy ring stretching modes; the peaks at 1182 and 883 cm^−1^ were related with the doping state of PPy, while that at 1046 cm^−1^ corresponded to the in-plane C–H deformation; the absorption bands at 940 and 957 cm^−1^ being attributed to deformation of polaronic and bipolaronic quinoid structure [44,45].

**Figure 5:**
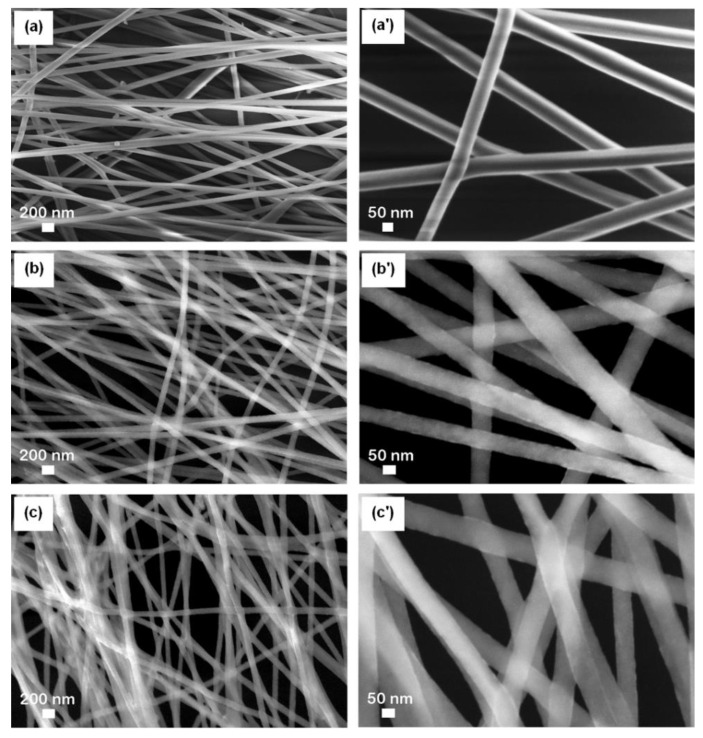
Representative FESEM images at different magnification of (**a**,**a’**) nylon, (**b**,**b’**) nylon-ZnO and (**c**,**c’**) nylon-ZnO/PPy, respectively.

A more detailed micromorphological characterization of fabricated materials was performed in order to highlight the formation of the hybrid fibrous organic–inorganic nanocomposite. Therefore, Figure 5 displays the FESEM images at different magnifications of nylon (Figure 5a,a’), nylon-ZnO (Figure 5b,b’) and nylon-ZnO/PPy (Figure 5c,c’), respectively. It can be observed that the fabrication steps did not alter the native morphology of nylon nanofibers and the high active surface of the electrospun meshes. The as spun nylon nanofibers had well defined shapes with an average diameter in the range of 70–90 nm, few nanofibers having even 50 nm. In contrast, after ZnO functionalization and thermal annealing, the inorganic compound fully covering as a very thin layer the electrospun networks. After this fabrication step, the nanofiber diameters could be found between 80 and 100 nm, so the ZnO thickness was estimated at about 10 ± 2 nm. The deposition of PPy onto core–shell nanofibers was homogenous, the PPy uniformly coating the ZnO layer and the diameter of the nanofibers increasing to 100–120 nm. In this case, the PPy film thickness was estimated about 20 ± 5 nm. After each deposition step, the nanofiber diameter lightly increased, which demonstrated the coverage of nets with both organic–inorganic compounds. All diameter estimations were in good agreement with TEM measurements.

**Figure 6:**
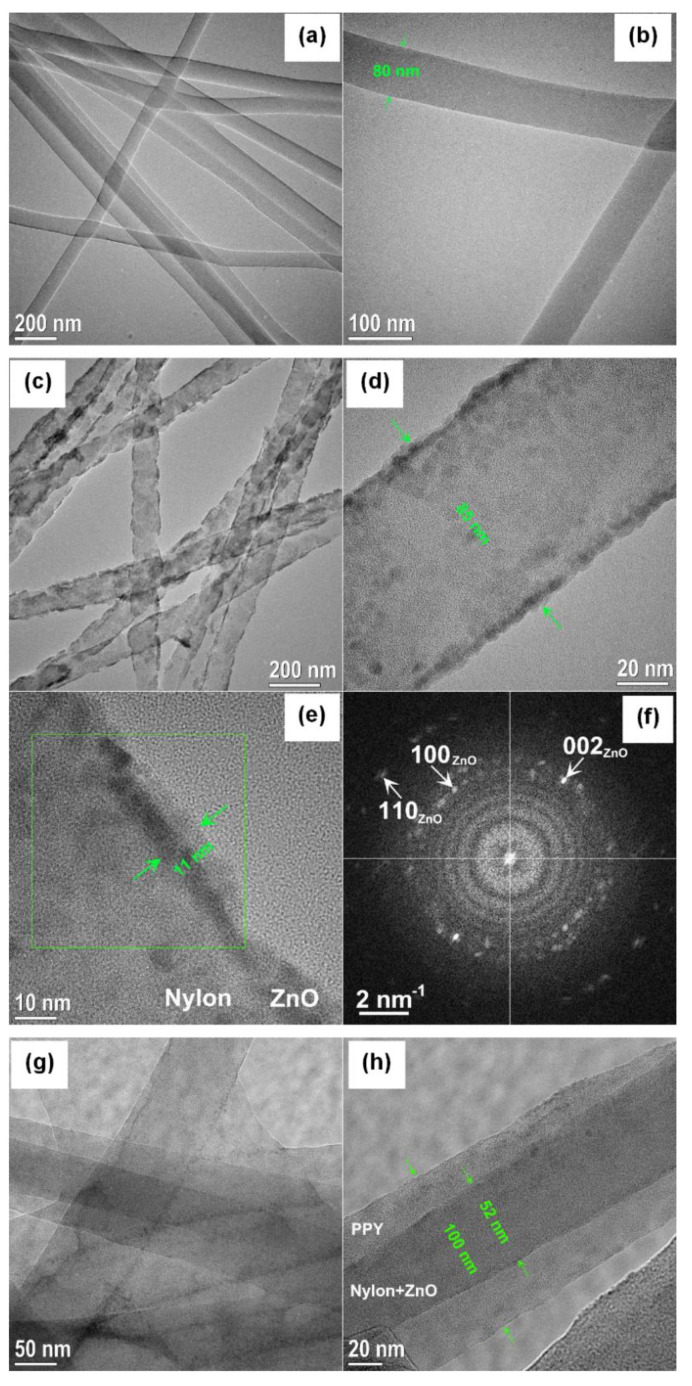
TEM images at different magnifications of (**a**,**b**) nylon, (**c**,**d**) nylon-ZnO and (**g**,**h**) nylon-ZnO/PPy; (**e**) high resolution transmission electron (HRTEM) image of an individual nylon-ZnO core–shell nanofiber and (**f**) FFT pattern corresponding to the area inside of green rectangle from the HRTEM image.

Figure 6a–d,g,h presents the TEM images at different magnifications of all prepared materials. As was emphasized by the FESEM images, the obtained nylon fibers were characterized by a well-defined shape with nanometric diameters (Figure 6a,b). The nylon-ZnO presents a core–shell like morphology (Figure 6c,d), which was very well evidenced in Figure 6d by the individual nanofiber analyze. The HRTEM image (Figure 6e) shows a continuous polycrystalline ZnO layer on top of nylon, having a thickness of about 11 ± 2 nm. The nylon-ZnO Fast Fourier Transformation (FFT) pattern (Figure 6f) corresponding to the area inside of the green rectangle from the HRTEM image revealed the formation of the hexagonal wurtzite phase of ZnO with P6_3mc_ space group by the presence of (100), (002) and (110) crystal planes [21]. These results were in a good agreement with XRD analysis. The electrodeposited PPy film was continuous (Figure 6g,h), the ZnO/PPy core–double shell thickness being around 24 ± 3 nm. The ZnO and PPy films were not separately viewed due to the sample high sensitivity at the electron beam.

## 4. Conclusions

Core–double shell nylon-ZnO/polypyrrole nanofibers were fabricated by functionalizing freestanding nylon 6/6 meshes obtained through electrospinning with ZnO by a sol–gel process and with polypyrrole by electropolymerization. It is worth mentioning that all synthesis parameters were optimized in order to cover only the nanofibers with very thin organic and inorganic layers, preserving the high active surface of the electrospun meshes. The presence of ZnO before and after polypyrrole deposition was evidenced by an elemental analysis carried out using EDX. The XRD pattern of ZnO coated nets validated the formation of ZnO in the hexagonal wurtzite crystal structure. From its reflectance spectrum a band gap of ~3.3 eV was estimated for ZnO. Both FTIR and Raman spectra confirmed the deposition of polypyrrole on ZnO coated networks. The detailed micromorphological investigation supported the hybrid nanocomposite fabrication mechanism and demonstrated that all deposition steps did not alter the native morphology of nanofiber webs. The very thin ZnO and polypyrrole films fully covered the nanofibers, the thickness of ZnO being estimated of about 10 ± 2 nm, while that of polypyrrole was about 20 ± 5 nm. The XRD and FESEM results were upheld by the TEM and HRTEM measurements. However, the versatility of the fabrication method allowed tailoring the material features according to targeted application. This kind of materials can easily find applications in many fields such as (bio)sensors, actuators, tissue scaffolds, etc., demonstrating their functionality being the topic of future work.
